# Addressing Kidney Transplant Shortage: The Potential of Kidney Paired Exchanges in Jordan

**DOI:** 10.1155/2024/4538034

**Published:** 2024-03-28

**Authors:** Mohammad H. Al-Thnaibat, Mohammad K. Balaw, Mohammed K. Al-Aquily, Reem A. Ghannam, Omar B. Mohd, Firas Alabidi, Suzan Alabidi, Fadi Hussein, Badi Rawashdeh

**Affiliations:** ^1^Department of Internal Medicine, Faculty of Medicine, Hashemite University, Zarqa 13133, Jordan; ^2^College of Medicine, Hashemite University, Zarqa 13133, Jordan; ^3^Jordan University of Science and Technology, Irbid, Jordan; ^4^Al Ain University, Al Ain, UAE; ^5^Department of Nephrology, Aurora Health Care, Milwaukee, Wisconsin, USA; ^6^Medical College of Wisconsin, Division of Transplant Surgery, Milwaukee, Wisconsin, USA

## Abstract

Jordan performed the Middle East's first living-donor kidney transplant in 1972. In 1977, the country became one of the first Arab countries to regulate organ donation and transplantation. Despite these early advances in living donor transplantation, Jordan's organ donation after brain death program remains inactive, making it challenging to meet organ demand and placing many patients on long transplant waiting lists. As of 2020, only 14.2% of the patients with end-stage kidney disease have access to a living donor. The scarcity of compatible living donors exacerbates Jordan's organ shortage, leaving patients with extended waits and uncertain transplant prospects. Due to the lack of living donors and the inactive brain death donation program, additional options are needed to meet organ demand. Kidney paired exchange (KPE), emerges as a potential solution to the problem of donor shortage and donor-recipient incompatibility. By allowing living donors to direct their donated organs to different compatible recipients, KPE offers the promise of expanding transplant opportunities for patients without suitable living donors. However, the current Jordanian law restricting living kidney donation to fifth-degree relatives further limits the pool of potential donors, aggravating the organ shortage situation. This article explores the feasibility of implementing KPE in Jordan and proposes an approach to implementing KPE in Jordan, considering ethical and legal aspects to substantially increase kidney transplants.

## 1. Introduction

Jordan set a crucial milestone in the field of transplantation in 1972 when it performed the first kidney transplant from a living donor, an unprecedented event in the Middle East [1]. The Jordanian parliament then took proactive action in 1977 by enacting laws governing organ donation and transplantation, becoming one of the first Arab nations to regulate such procedures [2]. Nevertheless, despite these early advances in living donor transplantation, the organ donation after brain death program in Jordan is not active, and the absence of an active brain death donation program significantly contributes to the difficulty of meeting the demand for organs, leaving many patients on long waiting lists with little hope of receiving a life-saving transplant [3–5].

**Figure 1:**
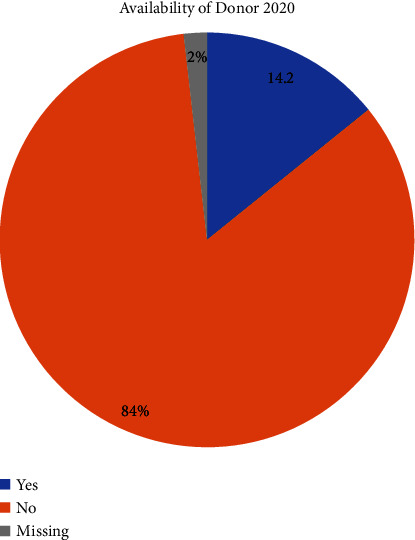
Distribution of end-stage renal disease patients based on donor availability in Jordan.

As of 2020, 2,598 patients in Jordan still need kidney transplantation and are deemed healthy enough to undergo the procedure. Out of those, only 368 (14.2%) had an accessible living donor [6, 7]. This stark disparity, where only a small fraction of patients have access to living donors, highlights the pressing challenge of organ shortage in Jordan. The number of patients in need of kidney transplantation continues to rise, while the availability of compatible living donors remains insufficient to meet the demand (Figure 1). From 2013 to 2021, there have been 1424 kidney transplantations in Jordan, only 8 of which were from deceased donors [8]. This reality places a significant burden on patients awaiting transplantation, with many facing prolonged and uncertain waits for a suitable organ match. The scarcity of living donors and the inactive status of the organ donation after brain death program further exacerbate the urgency of finding alternative solutions to address the growing demand for organs in the country.

Kidney paired exchange (KPE) also known as kidney paired donation was initially developed as a potential solution to the problem of incompatibility between donors and recipients [9, 10]. By allowing living donors to direct their donated organs to different compatible recipients, KPE offers a promising avenue to increase transplant opportunities for patients who lack compatible living donors. The implementation of KPE in Jordan could potentially provide a new and innovative approach to expand the pool of available organs and improve the chances of successful kidney transplantation for a greater number of patients in need. However, it is essential to acknowledge that the current Jordanian law restricts living kidney donation by requiring the donor to be no more than a fifth-degree relative of the recipient [1]. This legal constraint reduces the number of potential living donors for each patient, thereby aggravating the organ shortage situation. While the law was intended to protect against organ trafficking, it inadvertently limits the opportunities for living kidney transplantation for many patients who may not have suitable relatives within the required degree of relationship.

This work explores the possibility of implementing KPE in Jordan, the hurdles it may face, and the proposed benefits it may yield. We also proposed an approach to implementing KPE in Jordan, considering ethical and legal aspects to substantially increase kidney transplants. We hypothesize that a well-applied KPE program that considers global and Jordanian ethics and law and uses the most up-to-date software algorithm technology can significantly increase the total number of kidney transplants. This will reduce the morbidity and mortality caused by renal disease while awaiting kidney transplant and also solve the problem of demand and supply mismatch arising due to ABO and HLA incompatibility.

## 2. Matching Algorithm

**Figure 2:**
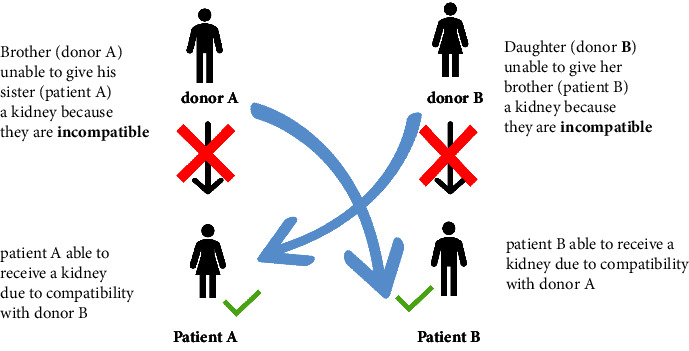
Simple two-way kidney paired exchange showing 2 donors (donor A and donor B) and 2 recipients (patient A and patient B); the red x is meant to show incompatible kidney (ABO or HLA incompatibility, for example) and the green arrow is meant to show compatible kidneys.

Kidney exchange, a mechanism allowing swaps and chains among patient-donor couples, aims to facilitate transplants for those with a willing yet incompatible donor. The concept of KPE was introduced by Rapaport in 1986 [11], but the first KPE took place in South Korea in 1991 [10]. The subsequent milestones include Europe's inaugural paired exchange in Basel, Switzerland, in 1999 and the U.S.'s first KPE in 2000 [12]. KPE can be set up as a three-way, four-way, or any n-way exchange [13, 14]. Two or more pairs can be organized for KPE [13, 15, 16] (Figure 2).

### 2.1. Traditional KPE

Traditionally, KPE demands incompatible pairs to locate other pairs with reciprocally matching incompatibilities. While it enables two ABO-compatible kidney transplants, finding such matching pairs can be challenging, especially with a limited pool. Two-way KPE, although facilitating, extends the matching duration and offers limited enhancement in transplant success rates [13, 17, 18]. Three-way exchanges, involving an extra incompatible pair, enhance transplant numbers and outcomes by eliminating the need for direct matching.

### 2.2. Domino-Paired Donation

**Figure 3:**
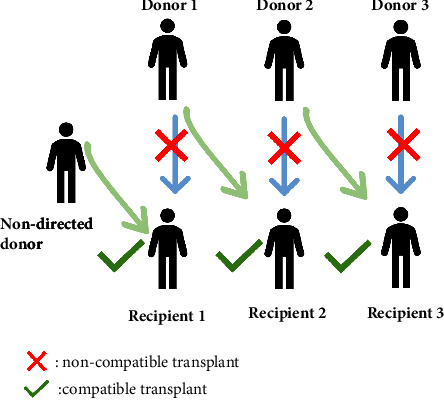
Chains of transplants initiated by nondirected altruistic donors, the domino effect.

Nondirected anonymous donors are individuals willing to donate a kidney without a designated recipient. These donors can initiate a KPD chain, potentially boosting transplant rates, especially for blood group O recipients [19, 20]. Here, the living nondirected donor kidney matches a recipient with an incompatible donor. This incompatible donor then donates to another compatible patient, creating a “domino effect” and leading to multiple live donor kidney transplants (Figure 3). This method can increase kidney exchange transplants by up to 20%. Benefits of domino-paired donation include expanding the blood type donor distribution, especially valuable for blood type O, and easing the matching requirements in KPD [21].

### 2.3. The Global State of KPE

Several countries around the world have successfully implemented KPE programs, which advanced the field of kidney transplant. Notable examples include United States, Canada, and India [22–24]. In these countries, they have set up innovative systems where different transplant centres collaborate to facilitate kidney exchange among incompatible donor-recipient pairs, which resulted in increasing the pool of donors and number of transplantations [25].

## 3. Promises and Challenges of KPE Program in Jordan

In Jordan, the adoption of KPE brings both promise and ethical quandaries. While KPE offers solutions to ABO and HLA incompatibility challenges, concerns arise around fairness, transparency, and potential gender disparities in transplantation outcomes.

### 3.1. ABO and HLA Incompatibility

**Figure 4:**
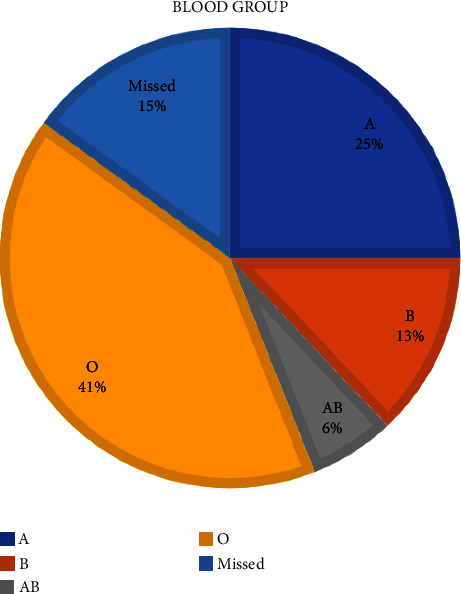
Distribution of blood groups among end-stage renal disease patients in Jordan in 2019.

**Figure 5:**
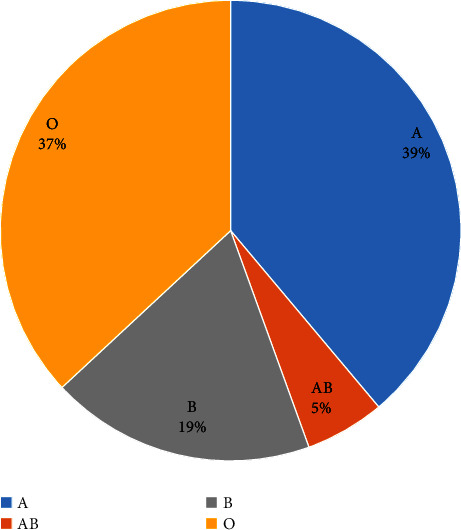
Distribution of renal transplants among jordanians (2013–2020) categorized by the recipient blood type.

The distribution of blood groups among ESRD patients in Jordan presents major challenges in the field of kidney transplantation (Figure 4). A significant barrier is ABO incompatibility, which occurs in 20–30% of the potential living-donor couples, limiting overall transplant options [26]. This is especially true for patients with blood type O, who account for 41% of all ESRD cases in the country [27] (Table 1) [28], (Figure 5). Unfortunately, these patients frequently struggle to find a match since type O donors are significantly underrepresented in KPE models. Recognizing this disparity, a three-way exchange program has been recommended [22]. This method, supported by effective results in numerous worldwide research studies, includes introducing another incompatible pair, increasing the likelihood of finding compatible pairings [17, 22]. The KPE program provides renewed hope to patients with the “difficult” blood type O by promoting KPE that increase their available donor pool. Given that blood type O recipients can only receive kidneys from donors with a similar blood type, employing kidney paired exchange (KPE) emerges as a strategic approach to optimize the allocation of type O kidneys. This methodology holds the potential to partially salvage type O kidneys by expanding the pool of available options. Specifically, individuals with blood types A, B, or AB become viable recipients, as they can receive kidneys from donors with blood types A, B, and A, B, AB, respectively. Consequently, KPE allows for wiser allocation of type O kidneys. Aside from ABO issues, the transplant community also faces HLA incompatibility. Even when a blood type match is found, this biological barrier can hinder effective transplantation. Studies to evaluate HLA polymorphism in Jordan and their effect on kidney transplantation are lacking. However, an Iranian study evaluated 512 kidney transplant recipients and found significant polymorphism in both class 1 and 2 antigens [29]. In a study done retrospectively at the King Hussein Cancer Centre (KHCC) to evaluate the cause of cancellation of potential kidney transplants, out of 642 possible donations, 143 were cancelled due to donor issue; out of those, 16 (11.2%) potential kidney transplants were cancelled due to ABO incompatibility, 10 (7.1%) other potential donors were positive for cytotoxic antibodies, only 3 (2.1%) had 0% HLA match, and only one (0.7%) potential donor had both cytotoxin antibodies and HLA incompatibility [3]. While desensitization has been proposed as one approach, it is associated with complications and risks [30]. In this context, KPE stands out as a ray of hope [26]. KPE can greatly improve the chances of finding a compatible donor-recipient match, not just based on blood type but also by figuring out the complicated HLA compatibility system since HLA matching provides much better outcome for kidney transplantation [31].

### 3.2. Gender Disparity

**Figure 6:**
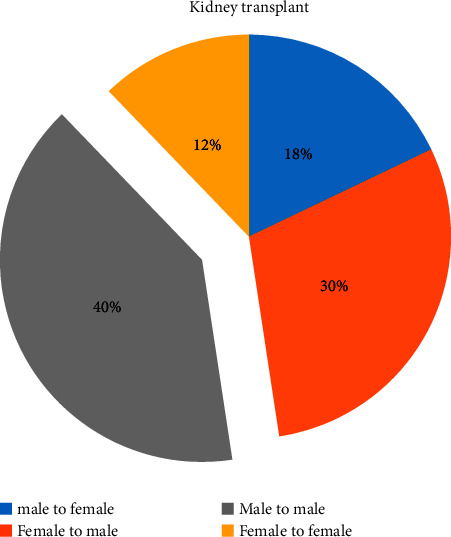
Distribution of kidney transplants in Jordan based on donor and recipient gender during 2013–2020.

Data from the Jordanian Centre for Organ Transplantation Directorate spanning 2013–2020 have highlighted a notable gender disparity in organ transplantation recipients. Of the kidney transplants for Jordanian patients, 17.9% were from male donors to female recipients, while 29.7% were from female donors to male recipients (Figure 6) which further elucidate this trend, showing that 40.2% of transplants occurred between males and 12.5% were female-to-female transplants [32].

This gender-based difference raises significant concerns about kidney transplant discrimination towards females in Jordan. In other countries, gender differences in organ donation seem to be also tilted in favor of males; in the USA, for example, according to the Organ Procurement and Transplantation Network data, women constitute only 38% of the transplant candidates on waitlists and during the duration of the study, and only 5.6% of women on dialysis received a kidney compared to 7% of men [33]. Women are also less likely to be referred for kidney transplant in southeastern states [34].

In Europe, information gathered from the European Society for Paediatric Nephrology/European Renal Association-European Dialysis and Transplant Association Registry, involving 6,454 patients across 35 countries, revealed that while medical aspects accounted for just 70% of the gender gap, there were additional factors influencing the difference. The lesser availability of preemptive transplantation for women stems from reasons that are partially connected to medical factors; other social and societal factors may explain this possible disparity [35].

The decreased frequency of female-to-female and male-to-female kidney transplants implies that immunological sensitization after childbirth prevents mothers from receiving kidney transplants. During pregnancy or labor, many mothers may have been sensitized to their own children, making it difficult for them to receive a kidney from a child. Due to immune incompatibility, this issue represents a significant barrier for mothers in urgent need of a kidney transplant, as their own children may not be suitable donors [33].

To address this important issue and improve transplant success for mothers, this cohort must be evaluated. The development of efficient treatments requires determining if the decreased rate of female-to-female transplants is related to immune sensitization or other social, financial, or medical factors. Furthermore, most Jordanian transplant centres only examine crossmatches, not HLA antibody specificities. This dearth of thorough testing may hinder the success of female-to-female transplants, resulting in lower graft and patient survival rates.

One other potential concern is the accumulation of highly sensitized recipients, particularly those with extensive exposure to HLA antigens through previous transplants or maternal exposure to fetal antigens. However, the expansion of the donor pool is a strategy to mitigate this issue. Highly sensitized patients often possess a broad range of HLA antibodies, making it challenging to find compatible donors in their families. Implementing of KPE for those patients make finding a suitable match much easier. In the case of O blood group recipients, their ability to receive kidneys from only O group donors limits their options.

This wise strategy not only enhances the chances of finding compatible donors for highly sensitized patients but also facilitates a fair and efficient distribution of available kidney donations. The inclusion of anonymous, nondirected donors would further improve this system. By doing so, it ensures that all transplant recipients, irrespective of their degree of sensitization or blood type, have better access to transplant opportunities.

In view of these challenges, adopting KPE program may be an alternative for resolving the gender discrepancy and improving transplant opportunities for mothers in Jordan. KPE allows living donors to donate organs to many compatible recipients, promising transplant prospects for those without compatible donors. By considering KPE, mothers with immunological sensitization concerns may have a better chance of receiving a compatible donor kidney. Thorough research is needed to implement KPE properly to enhance transplant outcomes and eliminate inequities for Jordanian female transplant candidates [21].

### 3.3. Ethical Concerns in KPE Program in Jordan

As with any novel approach to transplantation, the advent of KPE has been accompanied by ethical concerns. One critical concern pertains to the equitable distribution of organs. While this system offers hope for incompatible donor-recipient pairs, there is a risk of preferential treatment or unequal access, especially for vulnerable and marginalized groups. In Jordan, as much as one third of the population is living in abject poverty and about one quarter lacks medical insurance [36]. Inequality can manifest heavily in healthcare with factors such as the marital status, socioeconomic status (measured by wealth and education), access to the Internet, and geographical location playing significant roles in predicting the likelihood of not having health insurance. This can present a challenge to ensuring equal access to KPE in Jordan [36].

Informed consent becomes another ethical aspect, necessitating a clear understanding of the potential implications and risks for all involved parties in the paired donation program. The concept of “donation chains,” where multiple sequential transplants occur, poses further ethical dilemmas, potentially affecting the voluntariness and autonomy of donors down the chain. Barriers to donation included mistrust in the healthcare system and autonomy concerns. In Jordan, there already may be a lack of trust in the healthcare system due to the perceived lack of autonomy from potential donors [37], ensuring comprehensive regulations, transparency, and continuous monitoring to address these ethical complexities effectively remain paramount.

Another fundamental ethical issue which may appear is how to ensure that all patients' information regarding KPE remains confidential, especially when considering that during visits to healthcare professionals; only 18.1% of the general public were informed about patient privacy regulations [38]. 97% of those same respondents believed in patients' right to ensure data privacy before receiving medical care [38]. Much of the public in Jordan express apprehension about unauthorized access to electronic medical records and data breaches [38]. In Jordan, the electronic medical records (EMRs) are run by Hakeem® which is a nonprofit electronic system [39]. Therefore, protecting the sensitive medical information of donors and recipients is essential, and the procedure should adhere to medical ethics and data protection regulations.

Although attitudes and knowledge towards organ donation in Jordan are favourable [40], it is prudent to approach the issue of financial incentives and reimbursements for donors with caution from organ trafficking. In Jordan, there seems to be heightened concern towards receiving monetary reimbursement [37]. While it is permissible to provide financial assistance to cover medical costs associated with organ donation, it is crucial to avoid any form of organ exploitation or commercialization. Donors should be motivated by altruism, and monetary gain should not be the primary motivation for giving. The indicators for trafficking in human beings for the purpose of organ removal established by the EU act as a great source to help healthcare professionals and patients quickly recognize a potential organ trafficking crime [41].

More research is needed on how to invite participants in a way that does not put them under pressure and allows potential donors to enroll or decline freely. Another ethical concern is ensuring appropriate expectations and training patients and their families on how to handle possible negative outcomes relating to kidney transplantation that may still occur even with KPE.

### 3.4. Other Barriers

A notable barrier to consider in the context of KPE is the occurrence of reneging and the disruption of kidney donation chains. This issue has been extensively examined in a prior review article [42]. While only a small fraction, specifically 6 cases out of the 1748 paired donations, opted not to proceed with transplantation, their impact reverberates significantly along the subsequent links in the transplantation chain [43]. As this may pose a problem, certain precautions must be set in place to combat this. Having a team of experts on standby that can possibly link another chain of donors or to involve a nondirected anonymous donor to quickly bridge the gap that reneging may cause. While maintaining patient autonomy as the highest priority, explaining to donors and recipients the nature of KPE and that it involves chain of patients who will be affected as well has the potential to limit reneging.

## 4. Gradual Approach to Establishment and Expansion

The implementation of the KPE program in Jordan contains great potential for addressing the country's rising demand for kidney transplants. Donor-recipient matching in the diverse healthcare environment, which includes government, military, private, and university institutions, is complicated. Our suggested approach for establishing a KPE program in Jordan gradually emphasizes centralization, collaboration, ethical considerations, and strategic expansion.

### 4.1. Centralization and Standardization

The establishment of a National Transplant Centre appears to be an essential component of our proposed strategy. Centralization would provide a hub capable of coordinating all elements of kidney exchange, streamlining resources, and ensuring efficient and high-quality care delivery. The establishment of a National Transplant Centre to serve as the administrative hub for all transplantation efforts in Jordan will improve collaboration among healthcare institutions and transplant centres, fostering effective communication and resource allocation. The presence of the centre would promote data sharing and review, yielding significant data-driven insights on transplantation outcomes. Furthermore, a centralized approach would allow for protocol standardization and coordination, ensuring optimal patient outcomes and consistency of the KPE program. Furthermore, we are of the opinion that the collective cooperation of all centres in Jordan towards the establishment of a National Transplant Centre would expand the pool of potential organ donors and recipients, thereby enhancing the likelihood of an increase in the KPE transplant rate.

### 4.2. Ethical and Legal Foundations

The design and implementation of a KPE program in Jordan must comply with the highest ethical standards. Alignment with international principles, such as the Istanbul Declaration, will protect the interests and rights of donors and recipients. Building consensus among critical stakeholders, such as medical experts, government agencies, and healthcare organizations will boost the program's credibility and support. Engaging the public through targeted awareness campaigns will be critical to encouraging participation in KPE and educating the community about the advantages it provides, which will help extend the donor pool. Furthermore, building a dedicated social and legal team is critical for guaranteeing adequate education, eliminating organ trafficking, and implementing rigorous rules, all of which contribute to the program's integrity and ethical foundation.

### 4.3. Evaluation and Expansion

Continuous monitoring and evaluation will be critical in assessing the program's effectiveness and identifying areas for improvement. The analysis of transplant rates, graft survival, and patient quality of life will provide essential insights for optimizing the program's success. As the National Transplant Centre gains experience with two-way exchanges, a strategic expansion into more complex exchange types becomes feasible. This method of expansion ensures the program's adaptability and scalability without sacrificing the quality of service delivered.

## 5. Conclusion

The growing demand for kidney transplantation in Jordan, coupled with donor-recipient gender disparity and compatibility issues, necessitates a transformative solution. Our approach to instituting a KPE program in Jordan is a promising strategy for addressing these issues. By beginning with a centralized and standardized structure, we can establish a firm foundation for success in expanding kidney transplantation opportunities in Jordan by employing a measured and deliberate strategy.

The proposed program hopes to transform renal transplantation in Jordan by centralizing resources and standardizing protocols via the establishment of a National Transplant Centre. By encouraging collaboration among healthcare institutions and placing an emphasis on ethical considerations, the program ensures the legality and legitimacy of its operations. Furthermore, the establishment of this program would necessitate changing Jordan's law of transplant within the 5^th^ relative since this will be the most efficient step to expand the pool of donors for all recipients regardless of KPE; however, if coupled with KPE, this has the potential to exponentially increase the number of renal transplants done annually in Jordan. Continuous public engagement, meticulous evaluation, and a robust legal framework reinforce the foundation of the program. In addition, by rigorously adhering to international guidelines, the program addresses broader issues, such as organ trafficking and donor motivations, while maintaining the highest ethical standards. In the end, this transformative method not only offers a practical solution to the complicated medical problems of kidney transplantation but it also shows that Jordan is committed to addressing the fundamental human need for transplantation with compassion and ethical discipline.

## Figures and Tables

**Table 1:** Candidacy for transplantation among end-stage renal disease based on gender in Jordan.

Candidate	Gender		Total	%
	Male	Female		
Yes	1675	923	2598	35.6
No	2567	1754	4321	59.3
Unknown	231	140	371	5.1
Total	3881	2435	6316	100.0
